# Demonstration of an intrinsic circadian rhythm in bone resorption

**DOI:** 10.1038/s41598-025-16722-x

**Published:** 2025-09-25

**Authors:** Andrea L. Darling, Benita A. Middleton, Fatma Gossiel, Richard Eastell, Susan A. Lanham-New, Debra J. Skene

**Affiliations:** 1 Nutrition, Exercise, Chronobiology and Sleep, School of Biosciences, Faculty of Health and Medical Sciences, University of Surrey, Guildford, GU2 7XH UK; 2 The Mellanby Centre for Musculoskeletal Research, University of Sheffield, Sheffield, S10 2TN UK

**Keywords:** Bone turnover, Circadian, Collagen type 1 C-telopeptide, Type 1 procollagen N-terminal peptide, Constant routine study, Bone, Physiology

## Abstract

Daily 24 h rhythms in bone turnover have been demonstrated but whether these rhythms are intrinsically generated circadian rhythms is not known. We thus aimed to investigate this using the commonly used constant routine protocol where external factors such as meals, activity, sleep/wake and light/dark are kept constant. Serum procollagen type I N-terminal propeptide (sPINP) (marker of bone formation) and C-terminal telopeptide of type 1 collagen (sCTX) (marker of bone resorption), were measured in 2 hourly blood samples taken sequentially across 26 h in healthy individuals (n = 22, aged 19–33 years, 50% female). Concentration of sCTX showed a cosine rhythm in all males (acrophase (peak) time (mean ± SEM) 02:48 ± 14 h:min, amplitude 0.15 ± 0.02 ng/mL). All of the females had a statistically significant cosine + linear fit (acrophase 03:24 ± 20 h:min, amplitude 0.05 ± 0.01 ng/mL). There was no sex difference in acrophase, but females had a significantly smaller amplitude (*P* < 0.001). For sP1NP, only 4 males (36%) and 1 female showed statistically significant rhythms for either cosine, or cosine + linear models. Overall, sCTX, but not sPINP, exhibited a robust circadian rhythm in both males and females. This finding suggests that the circadian clock regulation of bone resorption by osteoclasts is robust, whereas circadian clock regulation of bone formation by osteoblasts is minimal.

**Supplementary Information:**

The online version contains supplementary material available at 10.1038/s41598-025-16722-x.

## Introduction

Intrinsic circadian rhythms are generated by the internal circadian timing system comprised of multiple clocks throughout the body. The internal circadian timing system is controlled by a central pacemaker in the brain’s hypothalamic suprachiasmatic nuclei (SCN). Circadian rhythms persist even when environmental influences are kept constant. The standard method for assessing intrinsic circadian rhythms in humans is the constant routine (CR) protocol^1^, whereby the external cues in the person’s environment are removed, minimised or kept constant in a highly controlled laboratory setting.

In contrast to circadian rhythms, diurnal or daily rhythms are changes over a 24-h day which are driven by a combination of exogenous environmental influences and intrinsic circadian rhythms. Potential environmental influences include, but are not limited to, the timing of light exposure, food, sleep and exercise. A recent systematic review^2^, as well as other research studies^3–5^ have suggested that human bone turnover has a circadian rhythm. In reality, however, these studies have only demonstrated diurnal rhythms since these studies did not control for all external factors (e.g. participants were ambulatory for part of the day, light levels varied, food was given as normal meals rather than hourly, sleep was permitted). Therefore, it is still unclear whether there is an intrinsic circadian rhythm in human bone turnover which is driving part of the observed diurnal variation.

The one exception to this was the study by St. Hilaire et al.^6^ that used a constant routine protocol and found a circadian rhythm in bone resorption by measuring urinary N-telopeptide (uNTX)^6^. However, since they did not report bone formation, it is still unclear whether there is a circadian rhythm in bone formation markers, as well as in the ratio between bone formation and resorption. In addition, the St. Hilaire et al. study^6^ was only conducted in women, and it remains unknown to what extent the acrophase and amplitude of the circadian rhythm of bone resorption would be different in men.

It is important to understand whether there is a circadian rhythm in human bone turnover, as observational evidence suggests a link between circadian misalignment, sleep deprivation and bone indices. For example, uncoupling of bone turnover, with a reduction in bone formation markers, has been found in humans with circadian disruption or sleep deprivation^7–9^. Moreover, observational studies have found a lower bone mineral density (BMD) in rotating shift workers^10^ and night-shift workers^11^ compared with day workers, which could be due to circadian misalignment and/or sleep deprivation. Although experimental studies have not found an acute effect of short term sleep restriction on bone turnover markers^12,13^, observational studies report a lower bone mineral density in sleep-deprived individuals compared to non-sleep—deprived individuals^14^. There is some evidence for an altered daily rhythm of bone resorption in osteoporosis^15^. Moreover, an association between genetic polymorphisms in circadian clock genes (e.g. PER1) and lumbar spine bone mineral density has been reported^16^.

In this current study, we aimed to assess whether there is an intrinsic circadian rhythm in both bone formation and resorption markers, in both women and men. We used banked blood samples from a constant routine study previously conducted at the University of Surrey^17,18^.

## Materials and methods

### Constant routine protocol

**Fig. 1:**
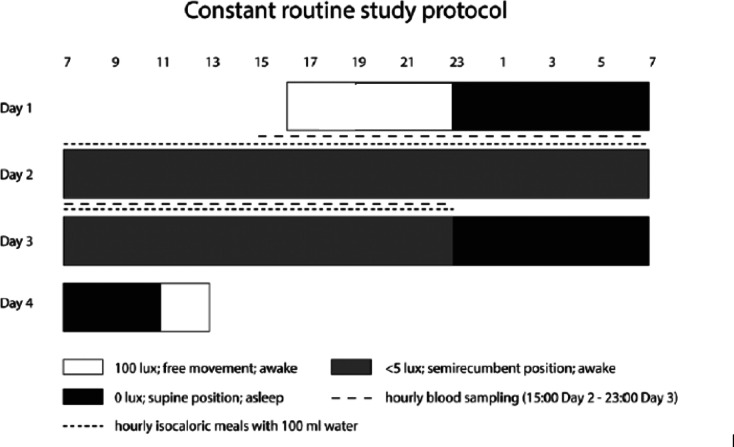
Study protocol for a participant with a sleep schedule 23:00–07:00 h. Grey bars represent controlled conditions of dim light (< 5 lx), semi-recumbent posture and wakefulness; black bars represent sleep opportunity in 0 lx; black lines represent hourly blood sampling and EEG recorded; black triangles represent standardised meals. Hourly isocaloric meals were provided from 07:00 h on day 2 until 23:00 on day 3. D = Day. Time of Day uses the 24 h clock. *Figure reproduced under permission from SAGE *via* STM permission agreement (*www.stm-assoc.org*). Original source: K. Lech, K. Ackermann, V.L. Revell, O. Lao, D.J. Skene, M. Kayser, Dissecting Daily and Circadian Expression Rhythms of Clock-Controlled Genes in Human Blood, J Biol Rhythms 31(1) (2016) 68–81.*

The full constant routine protocol has been described previously^17,18^ and is illustrated in Fig. 1. Briefly, the study was undertaken at the Clinical Research Centre at the University of Surrey, between January and August 2012. Participants were non-smoking, had no pre-existing medical conditions and did not use any prescription medications (except oral contraceptives). They had no history of drug or alcohol abuse. Participants were excluded if working night shifts or traveling across more than 2 time zones within 1 month of and throughout the study. Females were all oral contraceptive users and self-reported the brand and dose used. They had no history of menstrual irregularities.

In the 7 day lead up to the study, participants were asked to select an 8-h sleep period going to bed between 2200 and 0100 h and waking up between 0600 and 0900 h. They were required to obtain 15 min of outdoor light within 90 min of waking^17,18^. In the 72 h prior to the study, participants did not consume alcohol or caffeine and avoided bright light exposure and exercise in the evening^17,18^. Actiwatches (Cambridge Neurotechnology Ltd., Cambridge, UK), sleep diaries and the recording of wake time on a time stamped voicemail were used to assess compliance^17,18^.

The laboratory sessions ran from 16:00 h (Day 1) until 12:00 h (Day 4) (68 h in total). On day 2 the constant routine protocol commenced, participants awoke in dim light (< 5 lx in direction of gaze), at their normal wake time, in a semi-recumbent position. They continued to stay awake until 23:00 h on Day 3 when they started recovery sleep^17,18^. Throughout this period, participants consumed hourly isocaloric snacks with 100 ml of water and had no other food or drink intake. Blood samples were taken 2 hourly from 13:00 h on Day 2 until 23:00 h on Day 3 and the serum fraction was stored at − 80 °C.

### Measurement of bone markers

Measurement of serum collagen type 1 C-telopeptide (sCTX) and serum type 1 procollagen N-terminal peptide (sP1NP) was undertaken using the automated Cobas e411 electrochemiluminescent immunoassay (ECLIA) (Roche Diagnostics, Germany). Participants’ samples were randomly allocated to plate positions in terms of the participants’ sex. Sample timepoint was also varied in terms of plate position, reducing the risk of bias. Plasma melatonin and cortisol had previously been measured by radioimmunoassay (Stockgrand Ltd., University of Surrey, UK), the procedure for which has been previously described^17,19,20^. The assay limit of detection for melatonin was 3.9 pg/ml, with inter-assay coefficients of variation (CV) of 22.2% at 8.2 pg/ml, 10.4% at 33.2 pg/ml, 9.3% at 88.8 pg/ml and 9.0% at 121.4 pg/ml^17,18^. Equivalent data for cortisol were: assay limit 1.7 nmol/L and inter-assay CVs of 11.2% at 67.7 nmol/L, 11.0% at 520.0 nmol/L and 8.7% at 884.0 nmol/L^17^. Inter-assay CVs for the bone markers were 3.3% for sP1NP and 5.1% for sCTX.

### Statistical analysis

#### Power calculation

A power calculation was undertaken, based upon diurnal human plasma sCTX and sP1NP values. From previous data ^21^, using the peak to nadir difference in bone marker concentration, and the daily SD as a proxy for peak and nadir SD, for 80% power (based on repeated measures t-test between rhythm peak and nadir) we needed 11 participants. Specifically, for the sP1NP: sCTX ratio we needed < 6 participants; based on mean difference (MD) = 0.68, SD = 0.28, assuming nadir is 80% of the daily mean, peak is 40% above daily mean^21^. For sCTX we needed 11 participants; MD = 0.21, SD = 0.22 µg/L, assuming nadir is 75% of daily mean, peak is 40% above daily mean^21^. Therefore, out of the n = 30 participants available from the University of Surrey constant routine study, we selected n = 22 participants (n = 11 female and n = 11 male, aged between 19 and 33 years) who had blood samples at all time points.

#### Data analysis

A cut-off of *p* < 0.05 was used for all analyses. To prevent erroneous data points skewing the data, we corrected outliers for sP1NP and sCTX (11 out of 168 data points; see Supplementary File Tables S1-2 for details of each data point). Where possible, the datapoint was replaced with the average of the surrounding two data points, but if it was the first or last datapoint, it was removed completely from the analysis.

In addition, participant 910 (male) was slightly younger than the other participants and, as would be expected, had higher concentrations of sP1NP. Hence, for aggregated scores in the male group, this participant was included in the group Z score analysis for sP1NP and sCTX but was excluded from the group analysis of actual concentrations.

For melatonin, some values were below the detection limit (5 pg/ml), so were allocated as 3 pg/ml (i.e. half the detection value = 2.5 pg/ml, rounded up). For cortisol, no values were below the detection limit. For both melatonin and cortisol, datapoints where the sample was missing were denoted as blank.

#### Assessment of circadian rhythms

For each individual, we assessed the fit to: 1. A cosine curve (i.e. cosine with horizontal mesor (M3) compared to a horizontal line (M1), M3_M1) and 2. A cosine + linear curve (i.e. cosine with sloping mesor (M4) to sloping line (M2), M4_M2). We calculated acrophase and amplitude for each individual across all datapoints, as well as for within-sex groups. Each individual’s dim light melatonin onset (DLMOn25%; the time when melatonin reaches 25% of its peak value; abbreviated to DLMO) in the evening of Day 2 was used as a marker of circadian phase to assess the phase angle for sCTX and sP1NP relative to that of melatonin. Cosinor analysis^22^ was undertaken using MATLAB 2019 (MathWorks, Natick, MA, USA).

#### Ethics approval statement

The experimental protocols (constant routine study) were approved (given a favourable ethical opinion) by the University of Surrey (institution) (University of Surrey Ethics Committee EC/2011/127/FHMS). All procedures were conducted in accordance with the Declaration of Helsinki. Written and oral informed consent was obtained from the participants prior to any procedures being performed, and they were allowed to withdraw from the study at any time. All subject information was coded and held in strictest confidence according to the Data Protection Act (United Kingdom, 1998) and the General Data Protection Regulation (European Union/UK 2018).

## Results

### Descriptives

All data are mean ± SD unless otherwise stated. There were no statistically significant sex differences in age, body mass index (BMI), habitual bedtime, habitual waketime or Horne–Östberg score (Table 1). Average age for females was 24 (3.8) years (n = 11) and for males was 22.8 (2.4) years (n = 11). In terms of oral contraceptive preparations, n = 9 females took combined oestrogen and progesterone (Microgynon, Loestrin, Yasmin, Gedarel or Cilest), n = 1 was on progesterone only (Cerazette) and n = 1 had an unknown preparation.

**Table 1:** Participant characteristics. *A self-report questionnaire to estimate morningness—eveningness tendency.

	Females (n = 11)		Males (n = 11)		*P* value
	Mean	SD	Mean	SD	
Age (years)	24.0	3.8	22.8	2.4	0.39
BMI (kg/m^2^)	23.8	2.3	24.5	2.9	0.54
Bedtime (dec.h)	23.55	0.57	23.64	0.55	0.71
Waketime (dec.h)	7.55	0.57	7.64	0.55	0.71
Horne–Östberg score*	52.7	5.7	53.3	8.2	0.86

### Bone marker profiles

For reference, plots for each individual participant and the group mean (all Z scored, both clock time and DLMO corrected time), for sP1NP, sCTX and cortisol, are shown in Supplementary File Fig. S1-6. Mean (SD) for DLMO was 21.82 (0.98) h in females (n = 11) and 21.83 (1.19) h in males (n = 11) (Sex difference: *P* > 0.99).

**Fig. 2:**
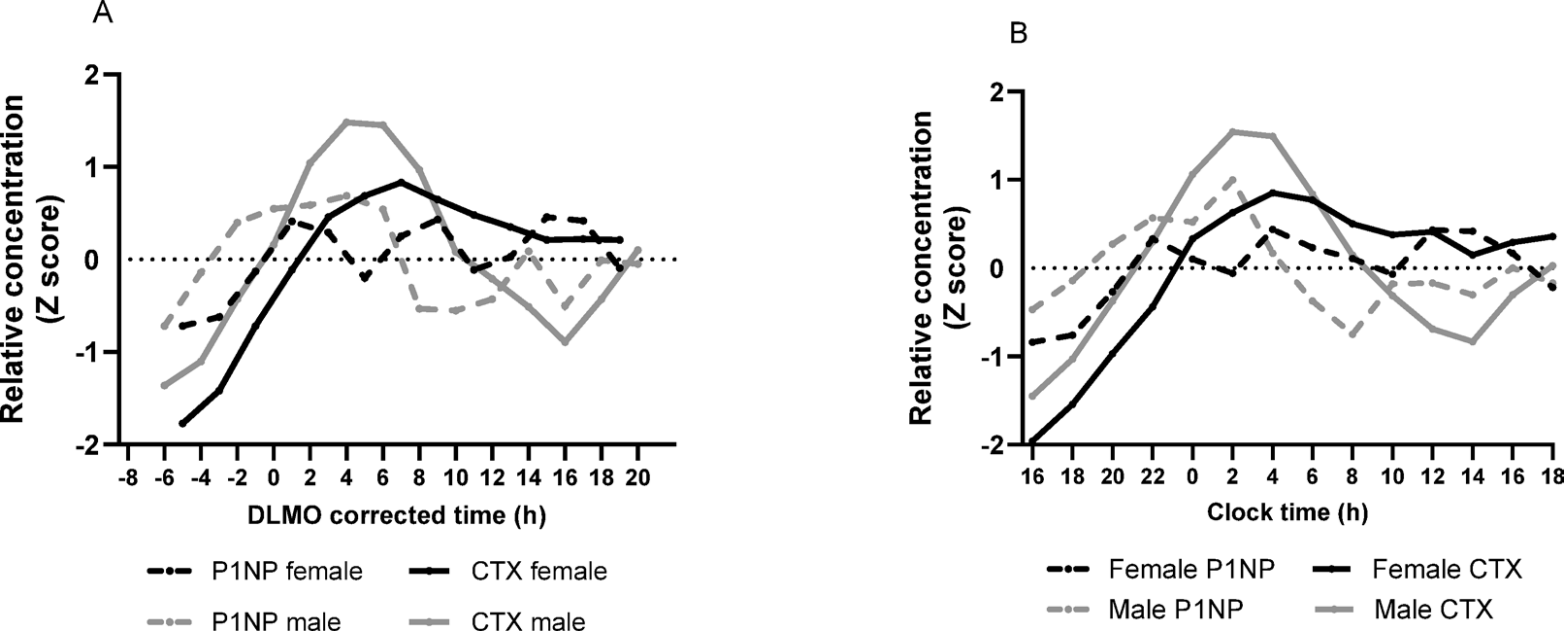
Group plots of sP1NP and sCTX by sex, for Z scores during a constant routine protocol, against DLMO corrected time or clock time. (**A**) mean Z scores by DLMO; (**B**) mean Z scores by clock time. Measurements were taken at 2 h intervals. DLMO corrected time (h) refers to the number of hours before or after DLMO. Mean (SD) DLMO was 22.0 h ± 1.3 h for the male group and 22.0 h ± 1.0 h for the female group.

**Fig. 3:**
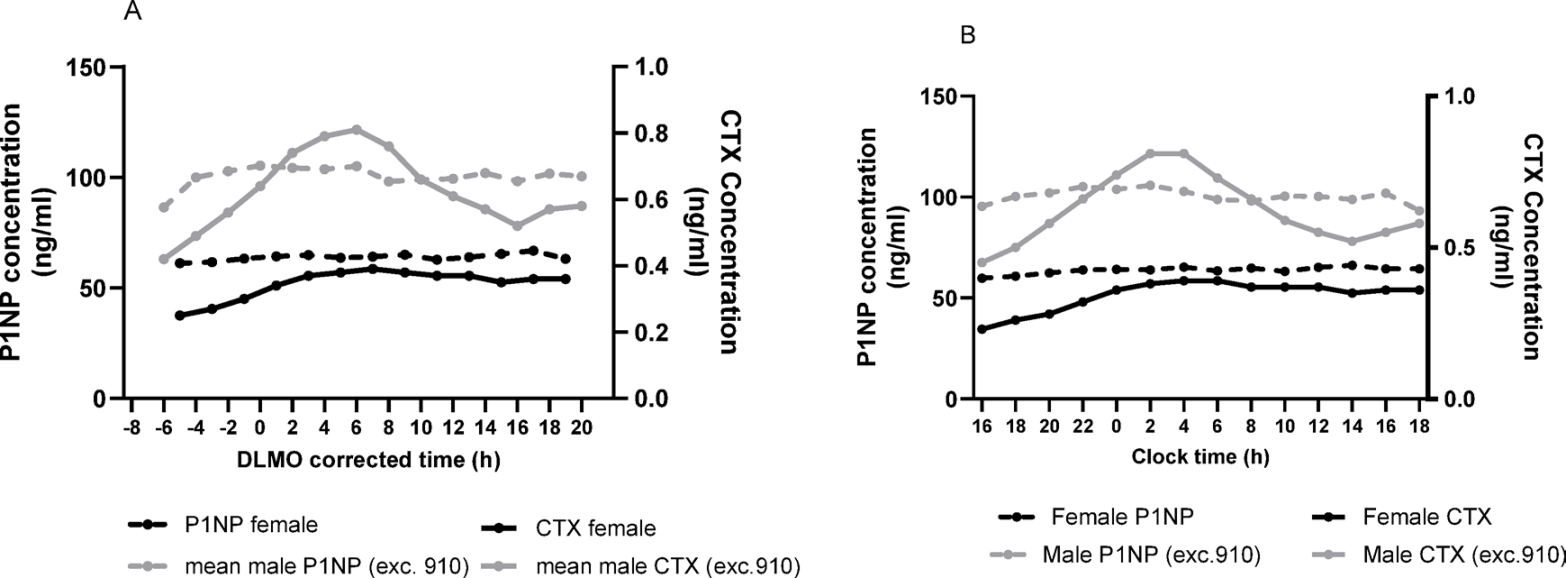
Group plots of sP1NP and sCTX by sex, for mean concentration (ng/ml) during a constant routine protocol, against DLMO corrected time or clock time. (**A**) Concentration by DLMO; (**B**) Concentration by clock time. Measurements were taken at 2 h intervals. DLMO corrected time (h) refers to the number of hours before or after DLMO. Mean (SD) DLMO was 22.0 h ± 1.3 h for the male group and 22.0 h ± 1.0 h for the female group.

Figure 2 and 3 shows the mean results by sex for sP1NP and sCTX, for both clock time and DLMO corrected time, expressed as Z score (Fig. 2) and raw concentration values (Fig. 3). As can be seen in Fig. 2A and B (Z scored data), males had a more pronounced rhythm in sCTX, with a peak at around + 4.5 h DLMO corrected time (02:30 h:min clock time), and a nadir at + 16 h DLMO corrected time (14:00 h:min clock time). For females, the rhythm was less pronounced, with a peak at + 8 h DLMO corrected time (06:00 h:min clock time), which then reached a plateau.

The sP1NP concentration profiles in both females and males are presented in Fig. 3A and B, respectively with females appearing to have a lower average concentration than males, but this sex difference was not statistically significant (see ANOVA results in next section of the manuscript). For the z-scored sP1NP data, there was some variation across time that appeared more evident in males than in females. However, no statistically significant rhythms were observed in the majority of the participants.

**Fig. 4:**
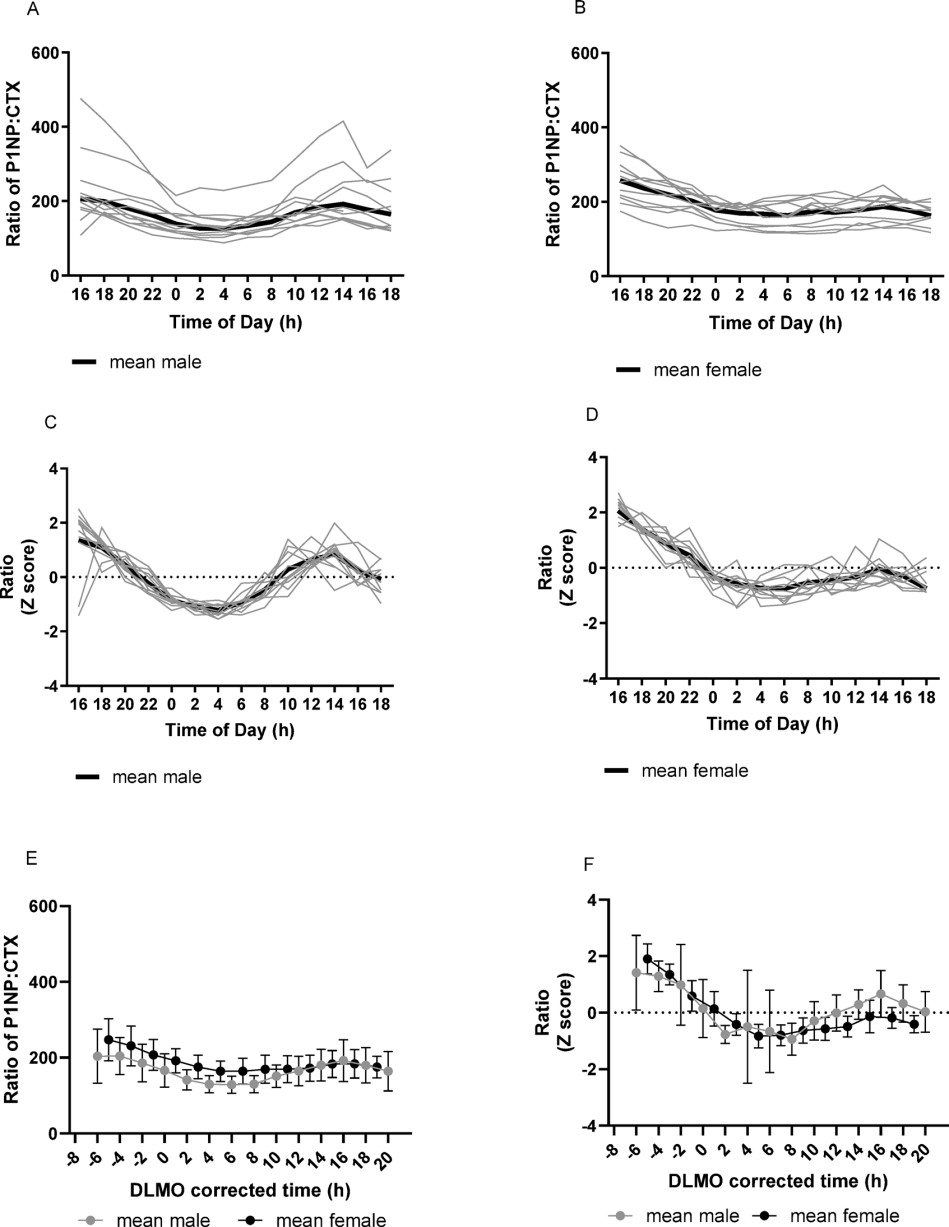
Individual and mean (± SD) plots of sP1NP:sCTX ratio by sex, for Z scores and concentration (ng/ml) according to time of day during a constant routine protocol. Measurements were taken at 2 h intervals. DLMO corrected time (h) refers to the number of hours before or after DLMO. Mean (SD) DLMO was 22.0 h ± 1.3 h for the male group and 22.0 h ± 1.0 h for the female group. (**A**) Ratio by Time of Day (h) males; (**B**) Ratio by Time of Day (h) females; (**C**) Z scored ratio by Time of Day (h) males; (**D**) Z scored ratio by Time of Day (h) females; (**E**) Ratio by DLMO for mean (± SD) male data and mean female data; (**F**) Z scored ratio by DLMO for mean (± SD) male data and mean female data.

For the sP1NP:sCTX ratio (Fig. 4A–F), for both concentration and Z-scores, there was a clear 24 h rhythm for both females and males, albeit this is likely driven by the sCTX rhythm, given the overall results of this study. For z scores in females, acrophase was at—7 h DLMO corrected time (15:00 h:min clock time) and amplitude was 0.94 (0.10) and in males, acrophase was at − 6.5 h DLMO corrected time (15:30 h:min clock time) and amplitude was 1.18 (0.38) .

**Fig. 5:**
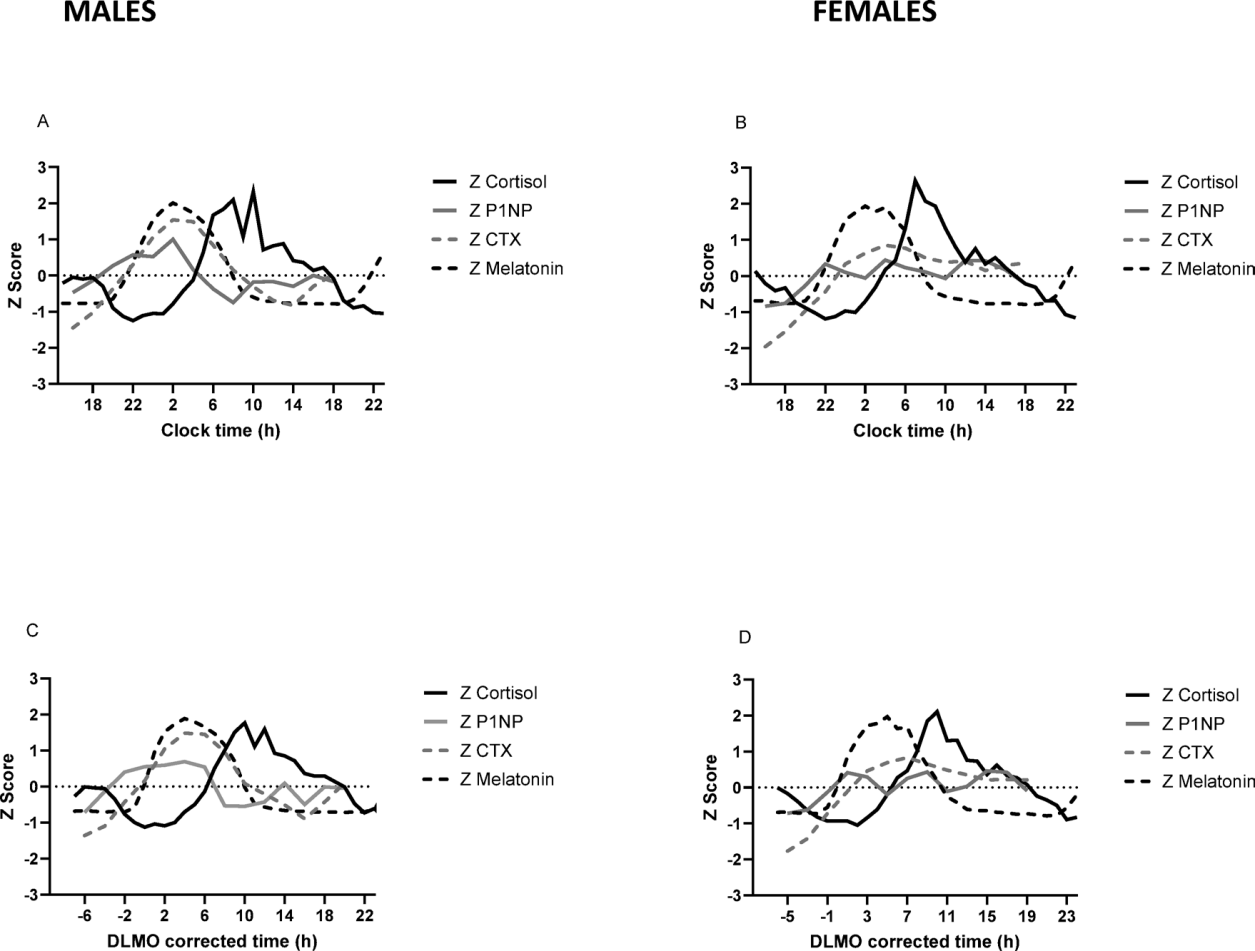
Group mean plots of Z scored cortisol, sP1NP and sCTX by sex, during a constant routine protocol. Measurements were taken at 2 h intervals (except for cortisol and melatonin which was hourly). DLMO corrected time (h) refers to the number of hours before or after DLMO. Mean (± SD) DLMO was 22.0 h ± 1.3 h for the male group and 22.0 h ± 1.0 h for the female group. (**A**) Mean Z scored data by clock time in males, for cortisol, melatonin, sP1NP and sCTX. (**B**) Mean Z scored data by clock time in females, for cortisol, melatonin, sP1NP and sCTX. (**C**) Mean Z scored data by DLMO in males, for cortisol, melatonin, sP1NP and sCTX. (**D**) Mean Z scored data by DLMO in females, for cortisol, melatonin, sP1NP and sCTX.

For z scored concentration data comparing cortisol and bone markers (Fig. 5A–D), for males (Fig. 5A and C) the peak of the CTX rhythm was 7 h earlier than cortisol (CTX around + 4 h DLMO (02:00 h:min clock time), cortisol peak around + 11 h DLMO (09:00 h:min clock time). The nadir of the cortisol rhythm was 14 h earlier than CTX (cortisol nadir around + 2 h DLMO (00:00 h:min clock time) and CTX around + 16 h DLMO (14:00 h:min clock time)). Therefore, in males, the CTX rhythm was around 7–14 h different in phase from cortisol, depending on whether you assessed the peak or nadir.

For females (Fig. 5B and D), cortisol showed a clear rhythm, peaking at + 10 h DLMO (08:00 h:min clock time) with a nadir at DLMO of 0 h (22:00 h:min clock time). However, in females, sCTX but not sPINP showed a discernible rhythm.

### Associations of clock time and sex with sP1NP and sCTX

For sCTX concentration (n = 9 males and n = 10 females), mixed between-within ANOVA showed a statistically significant clock time and sex interaction (*P* = 0.017), as well as for main effects for sex (*P* = 0.003) and clock time (*P* = 0.01), with plots showing that males might have a more pronounced sCTX peak compared with females. Similarly, for the sP1NP:sCTX ratio (concentration; n = 9 males and n = 9 females)), there was no statistically significant clock time and sex interaction (*P* = 0.05) or main effect of sex (*P* = 0.36), but there was a main effect of clock time (*P* < 0.001). For sP1NP concentration (n = 9 males and n = 9 females), there was no clock time and sex interaction (*P* = 0.30), and no main effect of sex (0.10), but there was a statistically significant main effect of clock time (*P* = 0.018).

Therefore, both sP1NP and sP1NP:sCTX ratio varied over time but not sex (suggesting no sex difference). An interaction between clock time and sex was seen for sCTX, showing that the bone markers in the sexes might behave differently over time for this bone marker.

### Assessment of circadian rhythms

To investigate further the observations from the plots, cosinor analysis was used to detect circadian rhythmicity. Cortisol showed a significant cosine rhythm (*P* < 0.05, rhythmicity ≥ 40%) in all females (acrophase (mean ± SEM) 09:29 ± 12 min, amplitude 247.7 ± 21.3 nmol/L and all males (acrophase (mean ± SEM) 09:54 ± 50.4 min, amplitude 164.9 ± 19.7 nmol/L).

Serum CTX showed a significant cosine rhythm (*P* < 0.05, rhythmicity ≥ 40%) in all males (acrophase (mean ± SEM) 02:28 ± 14 h:min, amplitude 0.15 ± 0.02 ng/mL). All of the n = 11 females had a statistically significant cosine + linear fit, (acrophase 03:24 ± 20 h:min, amplitude 0.05 ± 0.01 ng/mL). This suggests a difference in the shape of the rhythm by sex, with females also having a statistically significantly smaller amplitude (*P* = 0.001) (females 0.05 ng/mL vs. males 0.15 ng/mL). However, an independent t-test showed no statistically significant sex difference in acrophase (*P* = 0.23). For P1NP, only 4 males (36%) and 1 female showed statistically significant rhythms for either cosine, or cosine + linear models, therefore the mean acrophase and amplitude were not calculated. This suggests either an absent, or weak, circadian rhythm for sP1NP in both females and males.

## Discussion

We found that sCTX (a marker of bone resorption), but not sPINP (a marker of bone formation), exhibited an intrinsic circadian rhythm in both males and females. This finding suggests that the circadian clock regulation of bone resorption by osteoclasts is robust, whereas circadian clock regulation of bone formation by osteoblasts is minimal. Our results show the intrinsic peak (acrophase) in sCTX to be around 03:00 h, 5 h after DLMO. This is two hours earlier than that reported in some diurnal, non-constant routine studies (05:00 h^23^). Of note, 03:00 h is also the peak time of melatonin synthesis, and approximately 1 h before the nadir for body temperature (04:00 h). Our estimate for the sCTX peak occurs in the middle of the biological night. One crucial activity undertaken during sleep is tissue repair, and it seems logical that 03:00 h would be the peak time when osteoclasts are most active in excavating damaged bone tissue. An increase in bone resorption at night may help maintain the serum level of calcium during a calcium fast.

Our sCTX findings support that of St. Hilaire et al.^6^ who reported a circadian rhythm in bone resorption in women, as assessed by urinary NTX, with an acrophase at 03:00 h, similar to the acrophase for sCTX in our study (3:00 h for males and 3:24 h for females).

We also measured bone formation (sP1NP), which was not assessed by St.Hilaire et al.^6^, but we did not observe a clear circadian rhythm. However, it could be that sP1NP is not a sensitive enough marker to be able to detect small circadian changes in bone formation.

It is unclear why there was a possible sex difference in the shape of the sCTX rhythm, with the female data better fitting a cosine-linear model, and the male data better fitting a clear cosine rhythm, with no linear slope. In females, the observed linear component may reflect the impact of a non-circadian process. It is possible that the lower concentration of bone turnover markers in females could be due to the use of oral contraceptives, or another unidentified factor.

In order to explain the observed rhythmicity, we cannot rule out that bone could be responding to another rhythmic hormone or metabolite ex bone tissue, rather than being an intrinsic rhythm in bone tissue per se. However, it is reassuring that bone tissue studies have observed expression of the CLOCK^24^ and BMAL 1^25^ genes, two genes known to be involved in the regulation of the circadian system.

In terms of strengths and limitations, to the authors’ knowledge, this is the first analysis to demonstrate an intrinsic circadian rhythm in sCTX in both men and women. Limitations include not having data for BMD (or other relevant bone indices), meaning it was not possible to assess whether the amplitudes observed are driven by underlying differences in bone structure. This is particularly important considering there was a difference found between males and females for sCTX, which may be at least partly driven by known difference between males and females for BMD. Similarly, it is a limitation that we do not have usual exercise levels for the participants, as this may affect levels of bone turnover markers^26^, and exercise differences between females and males may explain some of the sex-based differences observed in this paper. These are key points for assessment in future research. Another limitation was the fact that the study sample comprised only young, healthy individuals. Since we have established a circadian rhythm in sCTX in younger people, research is now needed to assess, using the same constant routine methodology, whether this circadian rhythm is present in older individuals with osteoporosis, compared to age and BMI-matched controls. This will enable an estimation as to whether the circadian rhythm in bone is deranged in osteoporosis. It will also be important to investigate whether this rhythm is disrupted in shift workers with circadian misalignment and in sleep-deprived individuals.

## Supplementary Information

Below is the link to the electronic supplementary material.

Supplementary Material 1

## Data Availability

The data sets generated and analysed in this study are available from the corresponding authors upon reasonable request and where informed consent allows.
