# Supplementary material for: Demonstration of an intrinsic circadian rhythm in bone resorption
Source: Sci Rep. 2025 Sep 25;15:32558. doi: 10.1038/s41598-025-16722-x (PMC12462502; doi:10.1038/s41598-025-16722-x)
Supplement: Supplementary file 1 — Supplementary Material 1 [file 41598_2025_16722_MOESM1_ESM.pdf]

Demonstration of an intrinsic circadian rhythm in bone resorption

A.L. Darling[1], F. Gossiel[2], B.A. Middleton[3], R. Eastell[2], S.A. Lanham-New[1] and D.J. Skene[3]

Supplementary Table S1: Data points corrected: (outside of 20% of previous timepoint)

Subject	Datapoint	Marker	Original Value	Original value as proportion of previous datapoint	New Value	Method*
**901m**	08:00	P1NP	33.61	0.44	81.86	Averaged
	02:00	CTX	0.32	0.63	0.53	Averaged
**902m**	0:00	P1NP	70.03	0.57	122.85	Averaged
	0:00	CTX	0.56	0.66	0.87	Averaged
**918f**	18:00 D2	P1NP	3.86	0.10	-	Dropped*
**919m**	18:00 D2	P1NP	86.34	0.53	-	Dropped
	20:00	P1NP	131.2	0.74	174.55	Averaged
	16:00 D2	CTX	0.02	0.02	-	Dropped
	18:00	CTX	0.39	19.5	-	Dropped
**920f**	18:00 D2	P1NP	167.8	2.18	-	Dropped
	18:00 D2	CTX	1.13	2.90	-	Dropped

*Value dropped if last value (or second to last value with last value already dropped). Averaged means average taken from previous and subsequent datapoints. D=day (as there are two 16:00 and 18:00 timepoints).

Supplementary Table S2: Data points considered but not changed as within 20% of previous timepoint (if present) D=day (as there are two 16:00 and 18:00 timepoints).

Subject	Datapoint	Marker	Original Value	Original value as proportion of previous datapoint	New Value	Method*
**904m**	16:00 D1	P1NP	38.24	(first point)	-	-
	16:00 D2	P1NP	52.4	0.90	-	-
**905m**	18:00 D1	P1NP	97.08	0.89	-	-
**919m**	02:00	P1NP	183.0	1.08	-	-
**926f**	16:00 D1	P1NP	31.47	(first point)	-	-
**927f**	22:00	P1NP	42.46	1.18	-	-
**928f**	02:00	P1NP	40.43	1.19	-	-
**901m**	16:00 D1	P1NP	42.78	(first point)	-	-
**909m**	18:00 D1	P1NP	68.43	0.93	-	-
**918f**	02:00	P1NP	33.4	0.93	-	-

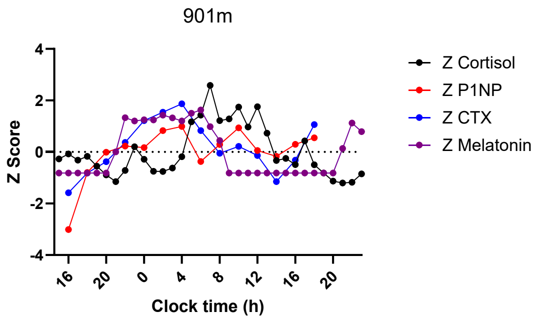
901m
Z Score
Clock time (h)
Z Cortisol
Z P1NP
Z CTX
Z Melatonin

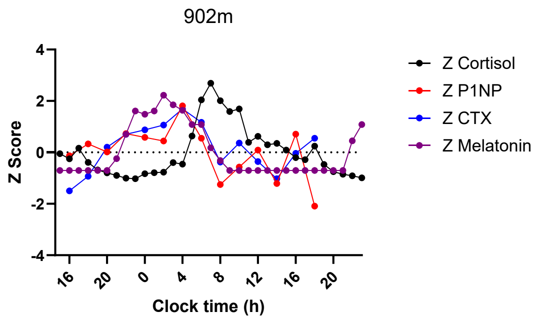
902m
Z Score
Clock time (h)
Z Cortisol
Z P1NP
Z CTX
Z Melatonin

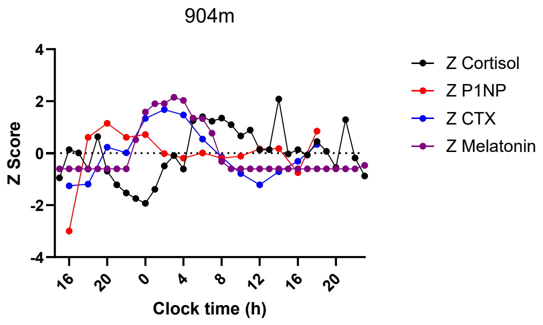
904m
Z Score
Clock time (h)
Z Cortisol
Z P1NP
Z CTX
Z Melatonin

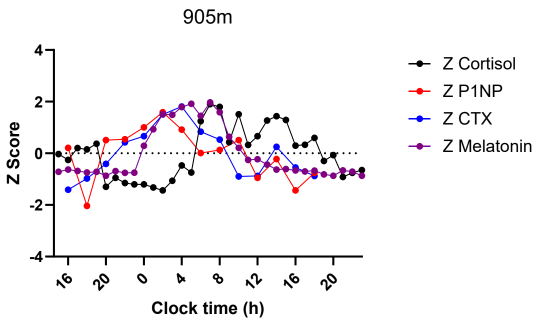
905m
Z Score
Clock time (h)
Z Cortisol
Z P1NP
Z CTX
Z Melatonin

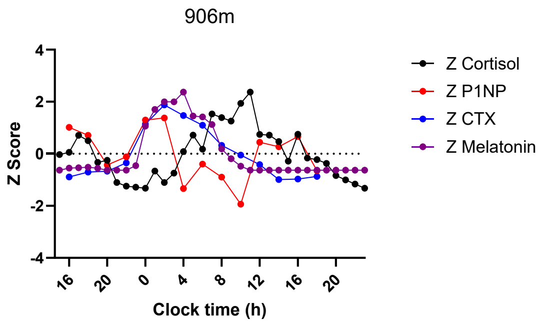
906m
Z Score
Clock time (h)
Z Cortisol
Z P1NP
Z CTX
Z Melatonin

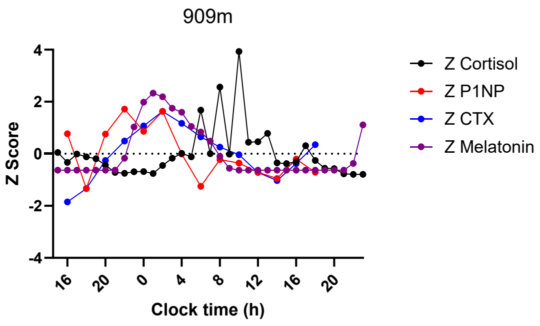
909m
Z Score
Clock time (h)
Z Cortisol
Z P1NP
Z CTX
Z Melatonin

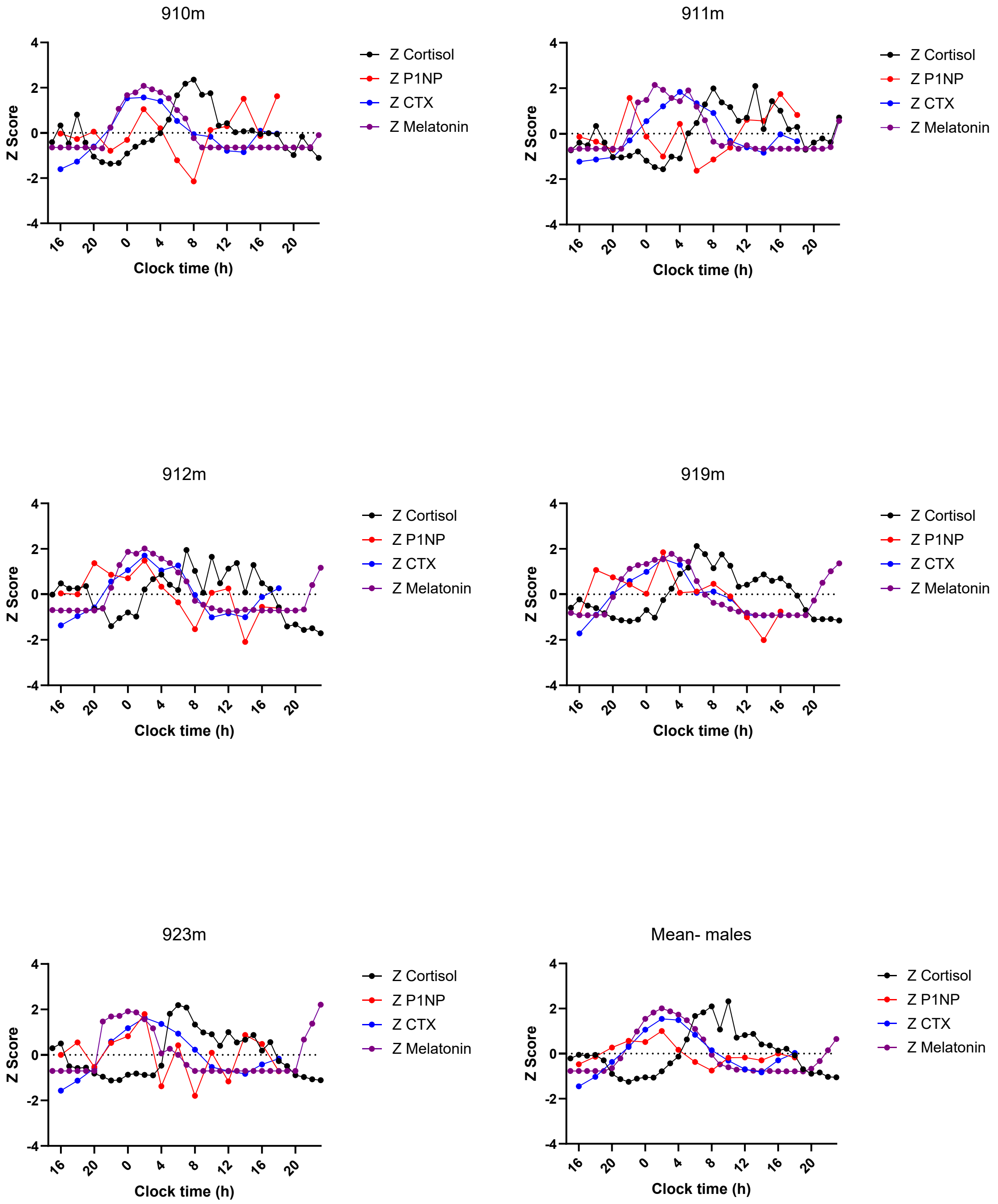

Supplementary Figures S1: Z scores for cortisol, melatonin, P1NP and CTX for each individual (male) and also for the group mean (998m, n=11)- time expressed as actual clock time.

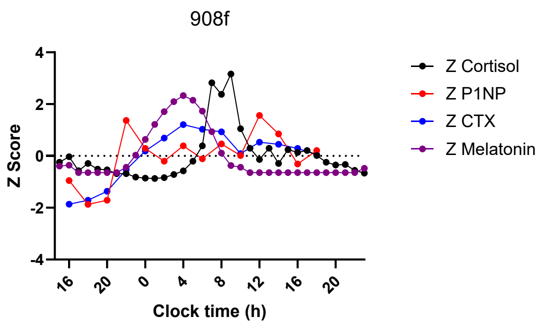
908f
Z Score
Clock time (h)
Z Cortisol
Z P1NP
Z CTX
Z Melatonin

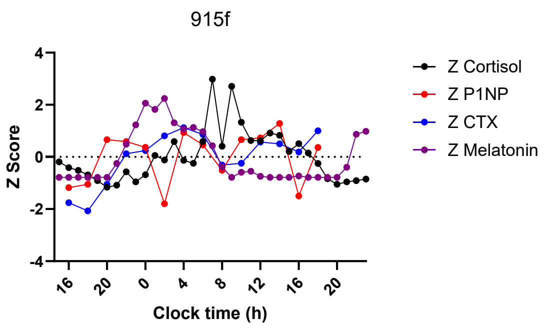
915f
Z Score
Clock time (h)
Z Cortisol
Z P1NP
Z CTX
Z Melatonin

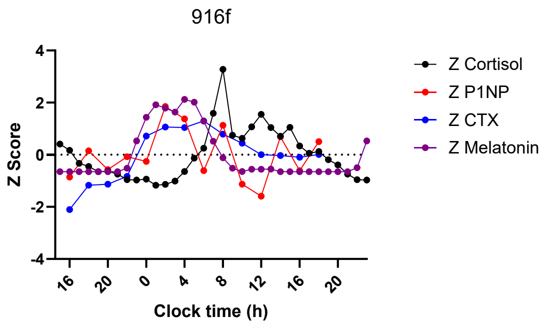
916f
Z Score
Clock time (h)
Z Cortisol
Z P1NP
Z CTX
Z Melatonin

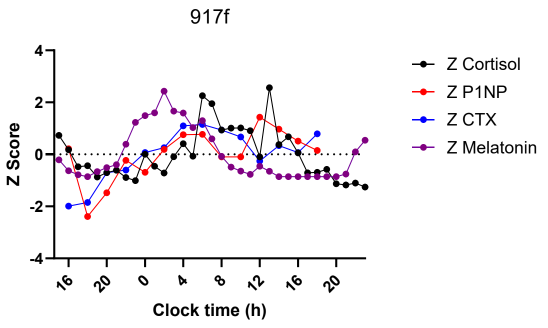
917f
Z Score
Clock time (h)
Z Cortisol
Z P1NP
Z CTX
Z Melatonin

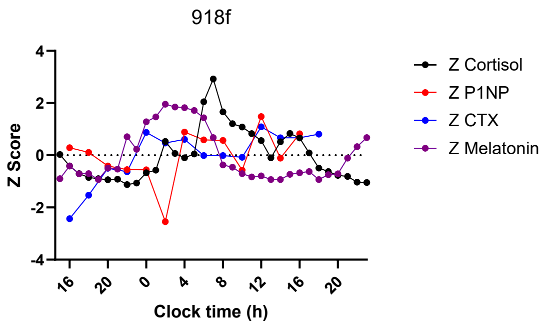
918f
Z Score
Clock time (h)
Z Cortisol
Z P1NP
Z CTX
Z Melatonin

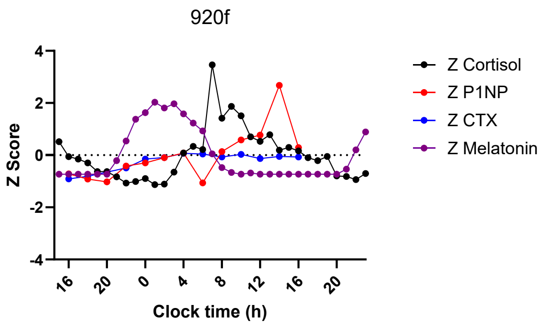
920f
Z Score
Clock time (h)
Z Cortisol
Z P1NP
Z CTX
Z Melatonin

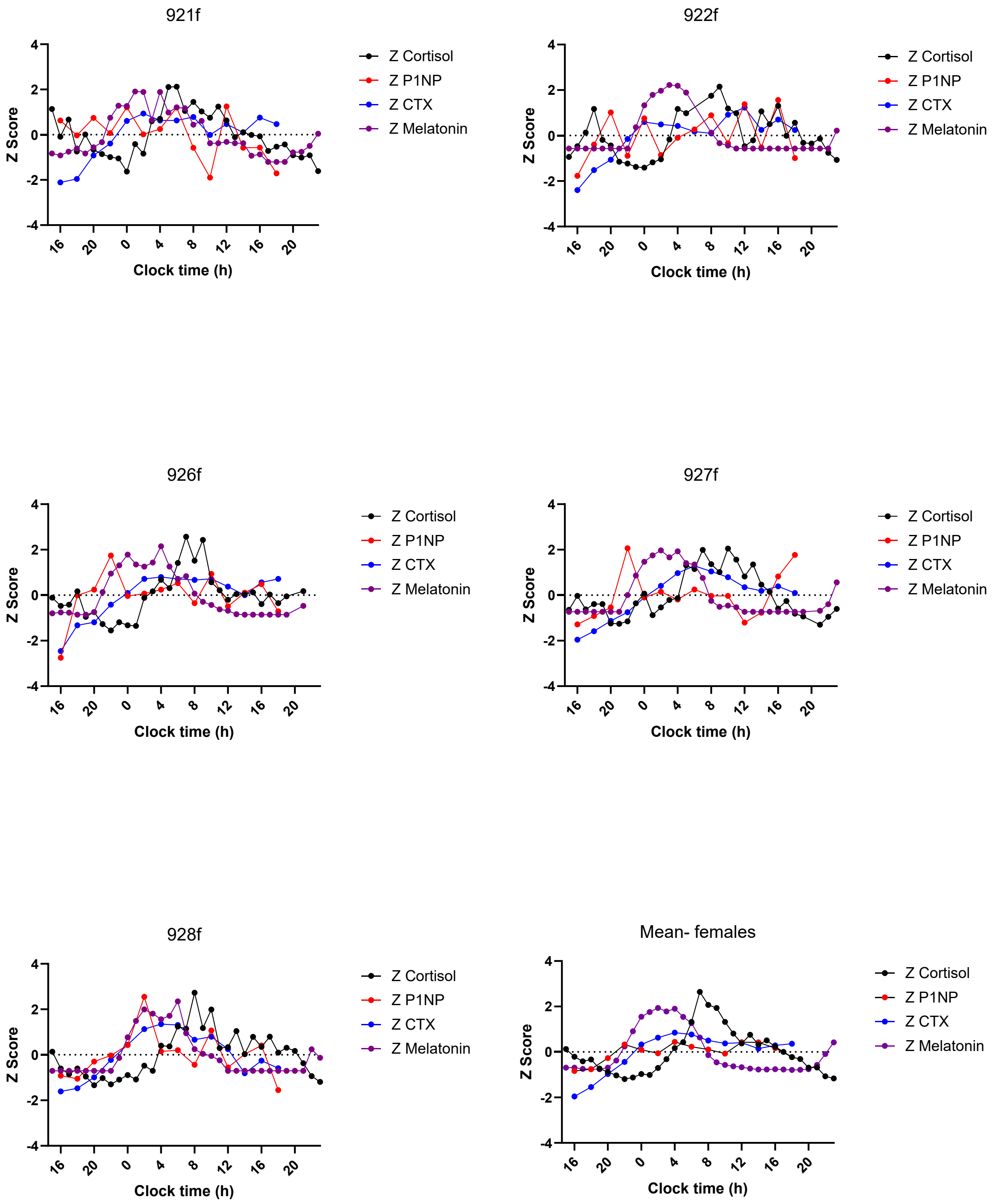

Supplementary Figures S2: Z scores for cortisol, melatonin, P1NP and CTX for each individual (female) and also for the group mean (999f, n=11)- time expressed as actual clock time.

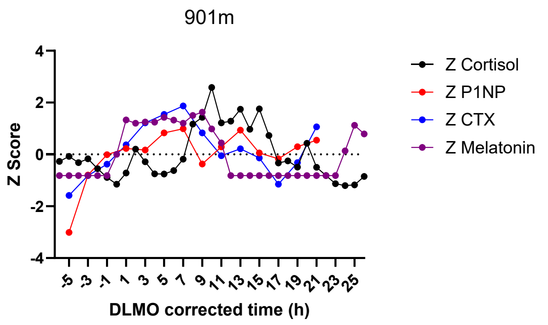
901m
Z Score
DLMO corrected time (h)
Z Cortisol
Z P1NP
Z CTX
Z Melatonin

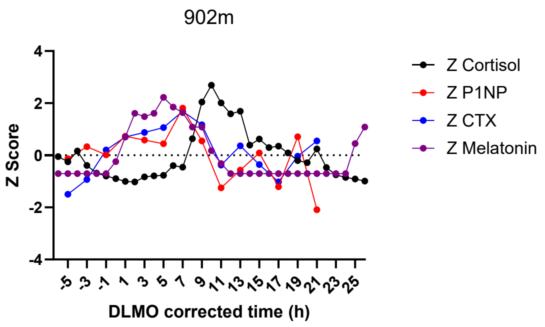
902m
Z Score
DLMO corrected time (h)
Z Cortisol
Z P1NP
Z CTX
Z Melatonin

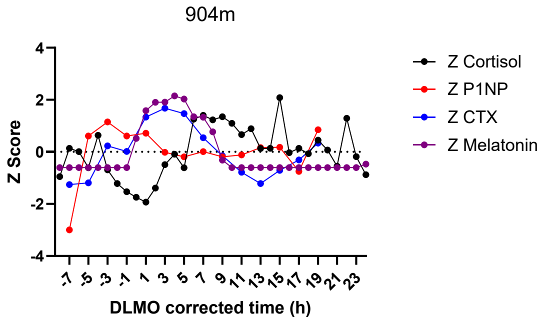
904m
Z Score
DLMO corrected time (h)
Z Cortisol
Z P1NP
Z CTX
Z Melatonin

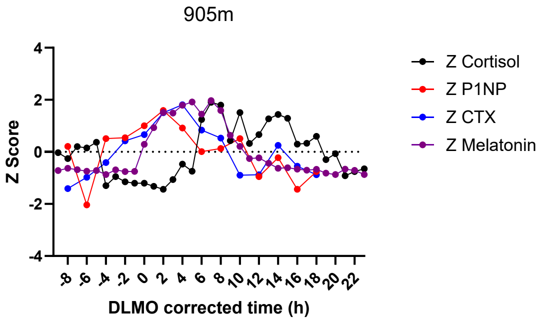
905m
Z Score
DLMO corrected time (h)
Z Cortisol
Z P1NP
Z CTX
Z Melatonin

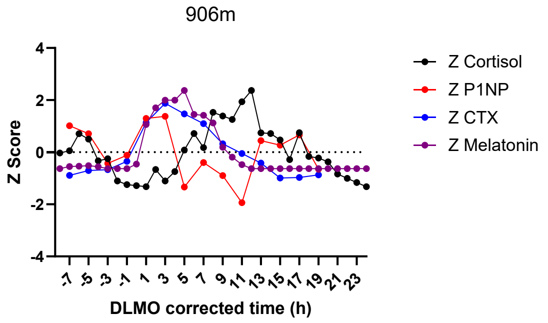
906m
Z Score
DLMO corrected time (h)
Z Cortisol
Z P1NP
Z CTX
Z Melatonin

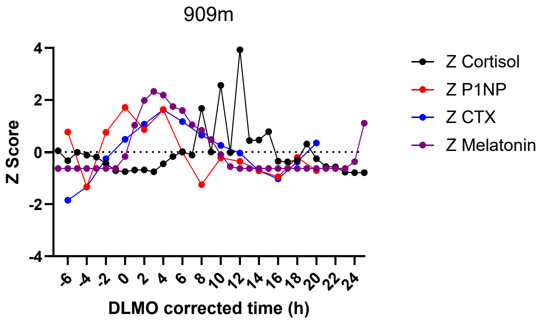
909m
Z Score
DLMO corrected time (h)
Z Cortisol
Z P1NP
Z CTX
Z Melatonin

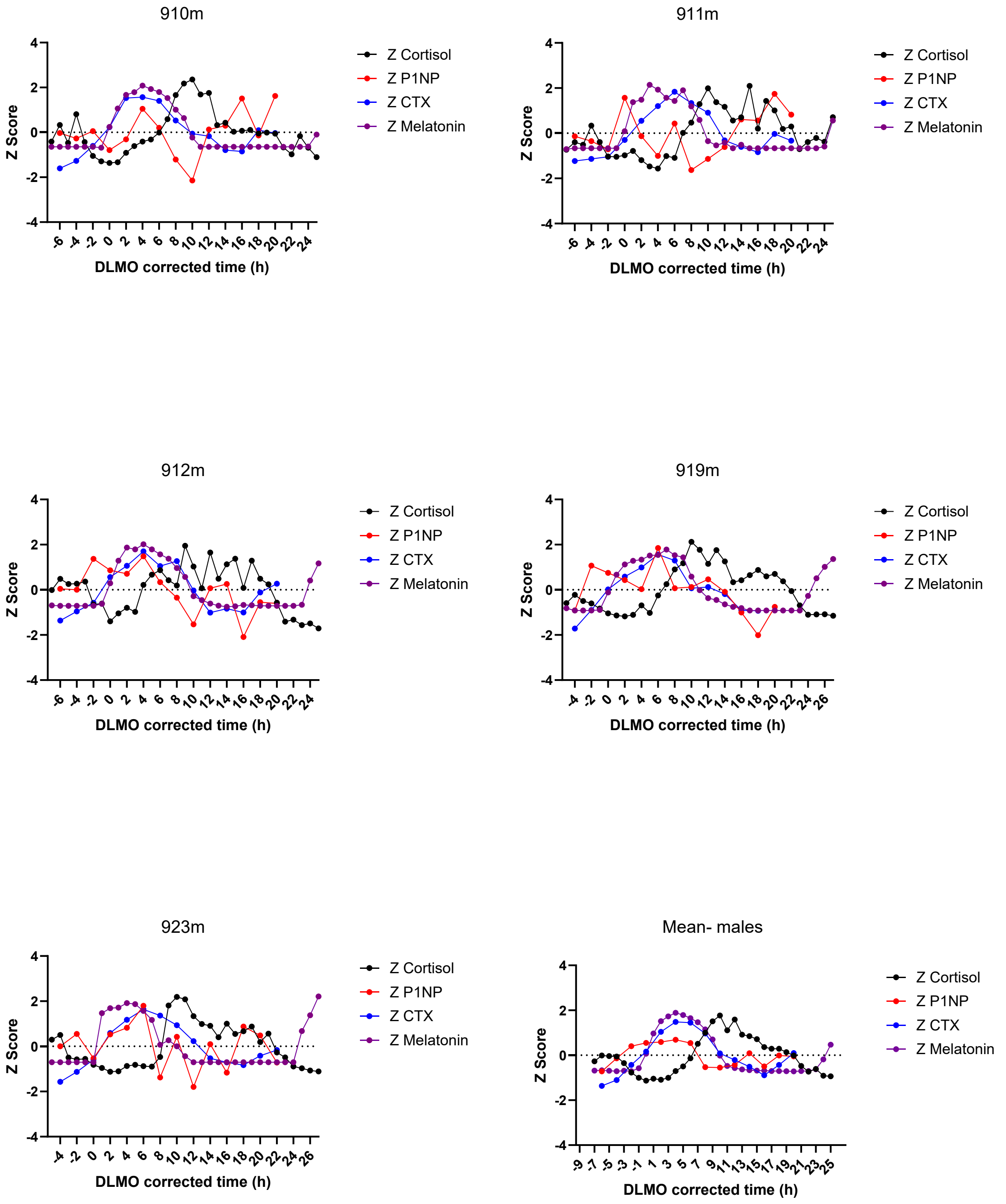

Supplementary Figures S3: Z scores for cortisol, melatonin, P1NP and CTX for each individual (male) and also for the group mean (998m for males, n=11)- time expressed as DLMO corrected time.

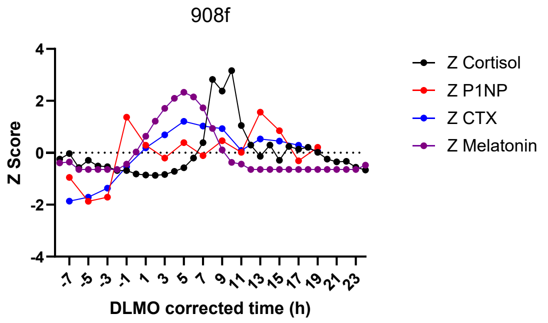
908f
Z Score
DLMO corrected time (h)
Z Cortisol
Z P1NP
Z CTX
Z Melatonin

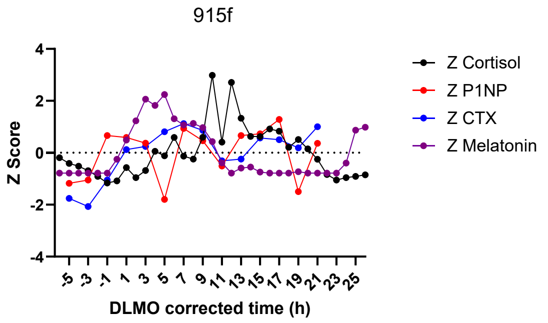
915f
Z Score
DLMO corrected time (h)
Z Cortisol
Z P1NP
Z CTX
Z Melatonin

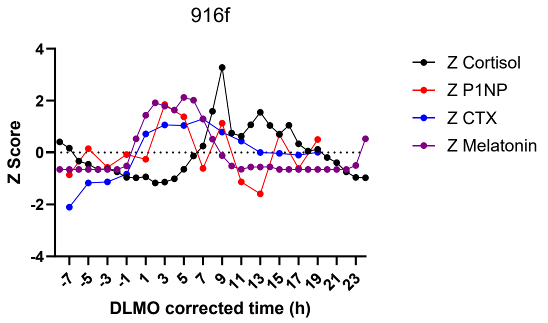
916f
Z Score
DLMO corrected time (h)
Z Cortisol
Z P1NP
Z CTX
Z Melatonin

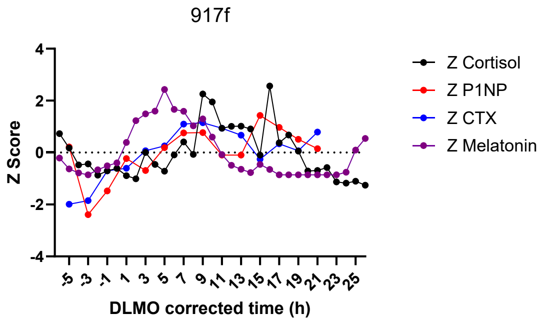
917f
Z Score
DLMO corrected time (h)
Z Cortisol
Z P1NP
Z CTX
Z Melatonin

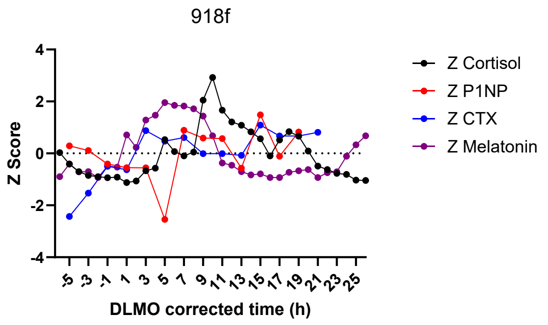
918f
Z Score
DLMO corrected time (h)
Z Cortisol
Z P1NP
Z CTX
Z Melatonin

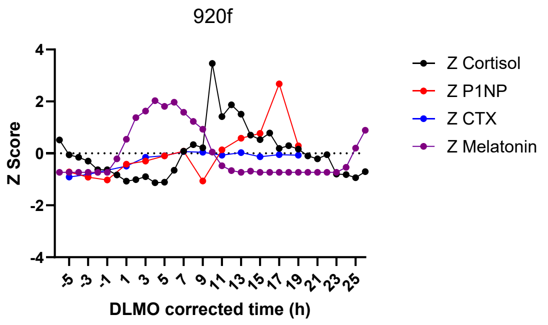
920f
Z Score
DLMO corrected time (h)
Z Cortisol
Z P1NP
Z CTX
Z Melatonin

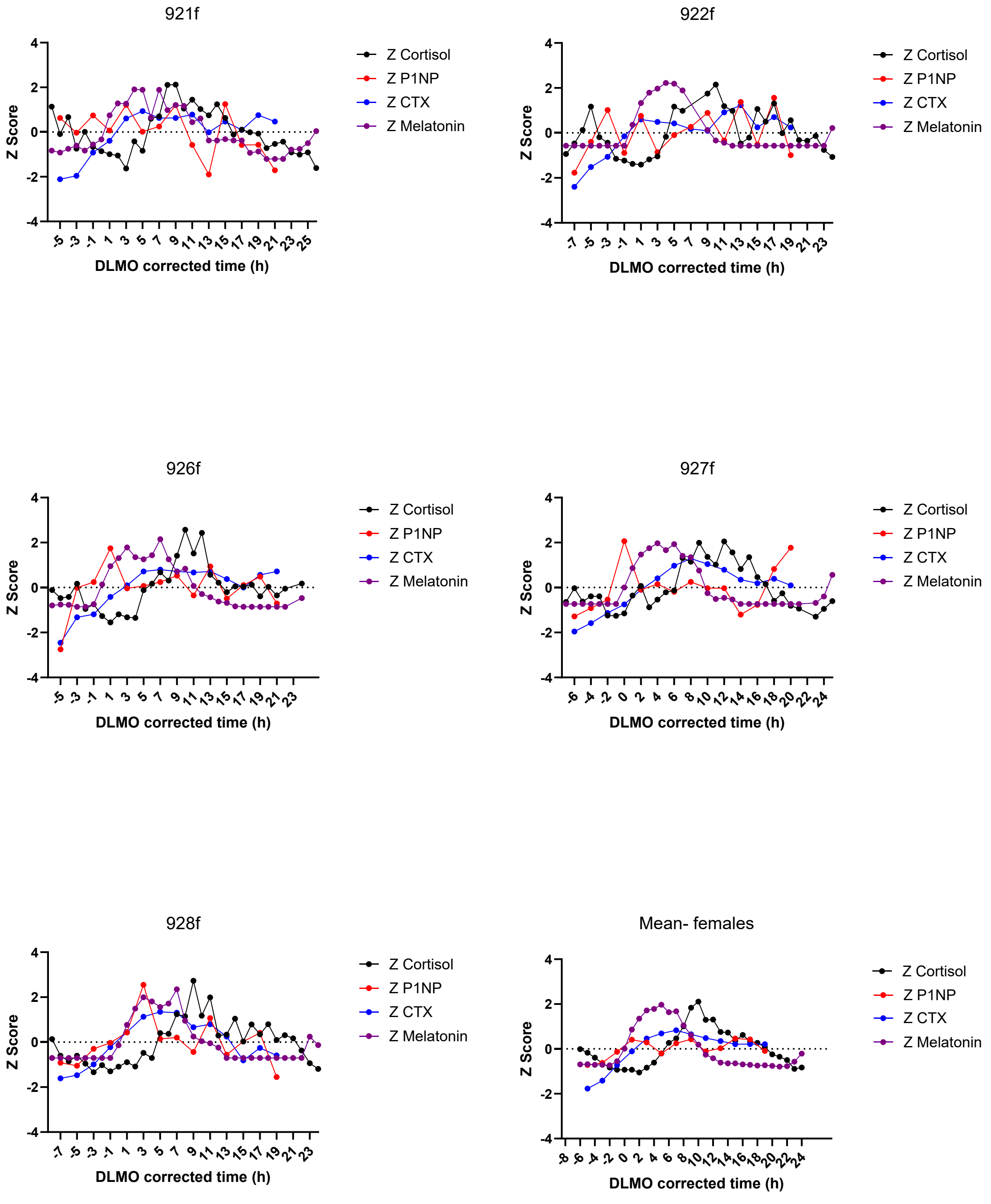

Supplementary Figures S4: Z scores for cortisol, melatonin, P1NP and CTX for each individual (female) and also for the group mean (999f, n=11) - time expressed as DLMO corrected time.

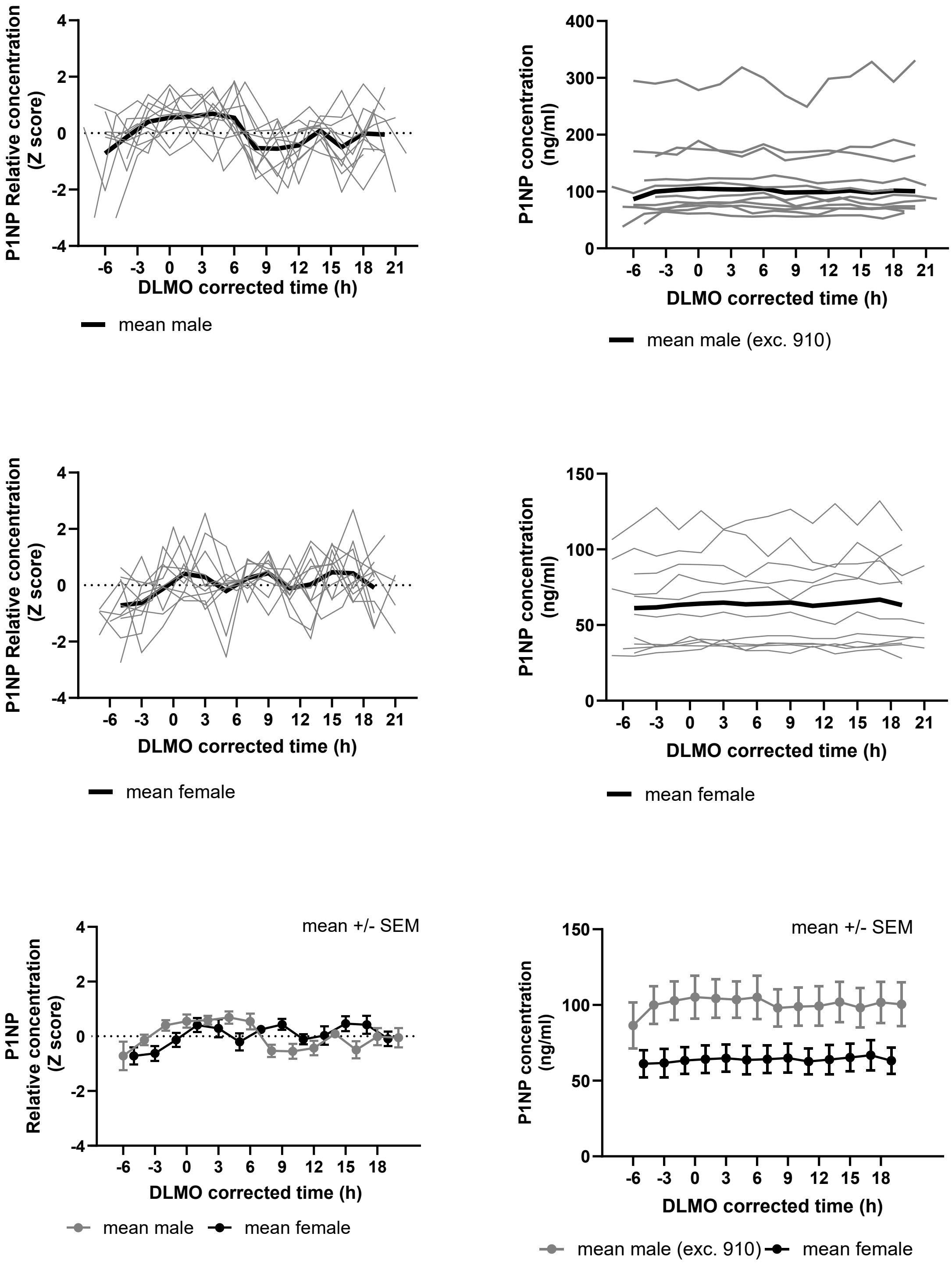

Supplementary Figure S5: Individual plots of sP1NP by sex, for Z scores and concentration (ng/ml) during a constant routine protocol, also showing group mean (± SEM). Measurements were taken at 2 h intervals. DLMO corrected time (h) refers to the number of hours before or after the dim light melatonin onset (DLMO). Mean (± SD) DLMO was 22.0 h ± 1.3 h for the male group and 22.0 h ± 1.0 h for the female group, but for individual participant data DLMO was individual specific.

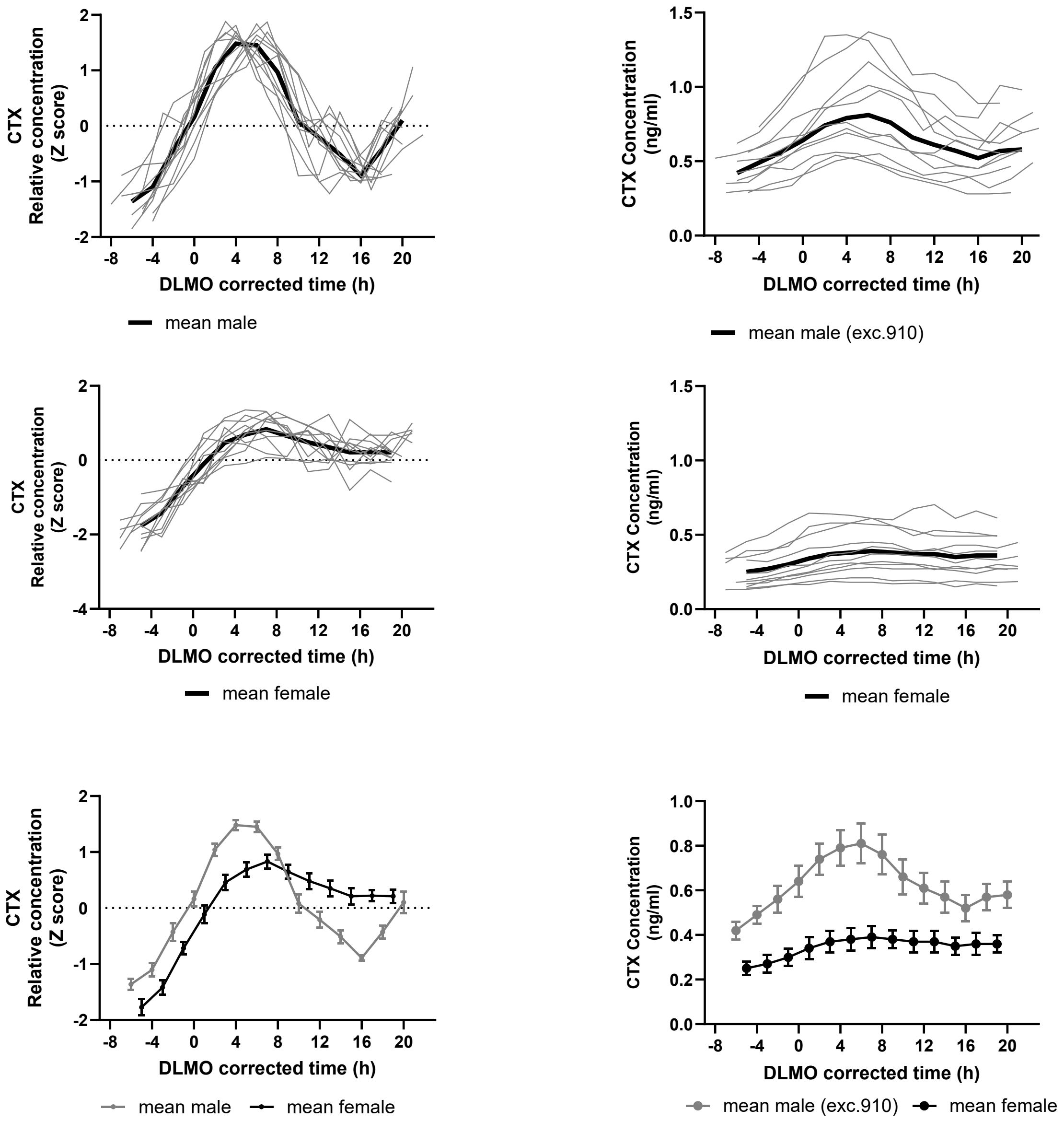

Supplementary Figure S6: Individual plots of sCTX by sex, for Z scores and concentration (ng/ml) during a constant routine protocol, also showing group mean (± SEM). Measurements were taken at 2 h intervals. DLMO corrected time (h) refers to the number of hours before or after DLMO. Mean (± SD) DLMO was 22.0 h ± 1.3 h for the male group and 22.0 h ± 1.0 h for the female group, but for individual participant data DLMO was individual specific.
